# The secretome of myocardial telocytes modulates the activity of cardiac stem cells

**DOI:** 10.1111/jcmm.12624

**Published:** 2015-07-14

**Authors:** Radu Albulescu, Cristiana Tanase, Elena Codrici, Daniela I Popescu, Sanda M Cretoiu, Laurentiu M Popescu

**Affiliations:** a Biochemistry-Proteomics Department, Victor Babeş National Institute of PathologyBucharest, Romania; b National Institute for Chemical Pharmaceutical Research & DevelopmentBucharest, Romania; c Division of Cell Biology and Histology, Carol Davila University of Medicine and PharmacyBucharest, Romania; d Department of Ultrastructural Pathology, Victor Babeş National Institute of PathologyBucharest, Romania; e Department of Advanced Studies, Victor Babeş National Institute of PathologyBucharest, Romania

**Keywords:** cardiac stem cells, telocytes, chemokines, cytokines, growth factors, SELDI-TOF, Luminex-xMAP, fibroblasts

## Abstract

Telocytes (TCs) are interstitial cells that are present in numerous organs, including the heart interstitial space and cardiac stem cell niche. TCs are completely different from fibroblasts. TCs release extracellular vesicles that may interact with cardiac stem cells (CSCs) *via* paracrine effects. Data on the secretory profile of TCs and the bidirectional shuttle vesicular signalling mechanism between TCs and CSCs are scarce. We aimed to characterize and understand the *in vitro* effect of the TC secretome on CSC fate. Therefore, we studied the protein secretory profile using supernatants from mouse cultured cardiac TCs. We also performed a comparative secretome analysis using supernatants from rat cultured cardiac TCs, a pure CSC line and TCs-CSCs in co-culture using (*i*) high-sensitivity on-chip electrophoresis, (*ii*) surface-enhanced laser desorption/ionization time-of-flight mass spectrometry and (*iii*) multiplex analysis by Luminex-xMAP. We identified several highly expressed molecules in the mouse cardiac TC secretory profile: interleukin (IL)-6, VEGF, macrophage inflammatory protein 1α (MIP-1α), MIP-2 and MCP-1, which are also present in the proteome of rat cardiac TCs. In addition, rat cardiac TCs secrete a slightly greater number of cytokines, IL-2, IL-10, IL-13 and some chemokines like, GRO-KC. We found that VEGF, IL-6 and some chemokines (all stimulated by IL-6 signalling) are secreted by cardiac TCs and overexpressed in co-cultures with CSCs. The expression levels of MIP-2 and MIP-1α increased twofold and fourfold, respectively, when TCs were co-cultured with CSCs, while the expression of IL-2 did not significantly differ between TCs and CSCs in mono culture and significantly decreased (twofold) in the co-culture system. These data suggest that the TC secretome plays a modulatory role in stem cell proliferation and differentiation.

## Introduction

Telocytes (TCs) are a particular type of interstitial cells that are distinguished by very long and thin cytoplasmic extensions with uneven caliber called telopodes (Tps) 1. Telocytes are best described in the heart 2–9, but they have also have been identified in the majority of mammalian organs, *e.g*. lungs 10, digestive tract 11–14, female reproductive organs 15–18 and placenta 19–21, urinary system 22,23, skeletal muscle 24 and neuromuscular spindles 25, liver 26–28, pancreas 29, salivary glands 30,31, skin 32–35, meninges and choroid plexus 36, eye 37, *etc*.

Cardiac TCs display distinctive features compared with other interstitial stromal cells (*e.g*. fibroblasts), such as extremely long prolongations – the telopodes – with unique ultrastructural morphology 1,38 and their immunophenotype; they are positive for CD34/vimentin and CD34/PDGFR-β 39. Mouse cardiac TCs spread and adhere more readily in culture compared with fibroblasts, which are less likely to spread irrespective of the substrate 40. In addition, the proteomic and gene profiles and miRNA imprints of TCs differ from those of fibroblasts, mesenchymal cells or endothelial cells 41–46. Moreover, TCs display distinct electrophysiological properties 47,48, and their endocytic properties along with their ability to release extracellular vesicles (EVs) contribute to intercellular information exchange and interactions 49. Telocytes have recently been shown to act as progenitor cells, especially during inflammatory/repair processes 50,51.

Despite the data that have accumulated since their discovery, the function of TCs has not yet been clearly identified. However, the most plausible assumption is that TCs are involved in intercellular signalling, during which they cooperate with stem cells 9,52. In the heart 53,54 and other organs, such as the lung 10, eye 37, skin 32 and skeletal muscle 55, TCs are closely associated with cardiac stem cells (CSCs) and accompany CSCs throughout their differentiation. Using electron microscopy and electron tomography, TCs were demonstrated to release three types of EVs: exosomes, ectosomes and multi-vesicular cargos 56. Moreover, the heart seems to feature a shuttle mechanism between TCs and stem cells, which involves bidirectional paracrine signals that are mediated by the EVs 9,57. Furthermore, TCs might play a role in the maturation of myocardial precursors into new cardiomyocytes in a normal heart or after ischaemic injury 53. Experimental infarction in rats equally demonstrated that TCs seem to be involved in neo-angiogenesis *via* the paracrine secretion of angiogenic microRNAs, VEGF or nitric oxide synthase (NOS2) in the infarction border zone during the late stage of myocardial infarction 58. Telocytes transplantation in the infarcted area and border zones improved cardiac function *via* factors and micro-vesicles secreted by the TCs 59,60.

The presence of regulatory molecules, cytokines, chemokines and growth factors in the CSC environment played crucial roles in stem cell growth and differentiation, especially in the heart 57. Therefore, we sought to analyse the secreted proteins (secretome) of TCs and assess its influence on stem cells. We also compared the secretomes of TCs and CSCs.

We report the protein/peptide secretory profile of TCs from rodent hearts, which confirmed the presence of VEGF, IL-6 and MIP-2 in the supernatants of TCs in culture. In addition, MIP-1α, MCP-1 and GRO/KC, along with several less abundant cytokines (IL-2, IL-5, IL-13 and IL-15), were identified. Our results indicate that TCs could sense and re-direct the cellular microenvironment to increase the renewal capacity of CSCs.

## Materials and methods

### Animals and ethics statement

Adult C57B6 male mice (C57BL/6; # 000664; The Jackson Laboratory, Bar Harbor, ME, USA) weighing 25–30 g (10–12 weeks old) and male adult Wistar rats weighing 150–200 g (8–10 weeks) were used in this study. The rodents were housed in air-conditioned rooms (22–24°C) under a 12-hrs light–dark cycle at a standard facility with *ad libitum* access to food and water. The rodents were killed by cervical dislocation and immobilized in the supine position with their necks extended. The thoracic cavity was then cut open to expose the hearts, which were consequently removed from the body.

This study was approved by the Bioethics Committee of ‘Victor Babeş’ National Institute of Pathology, Bucharest according to the institutional guidelines and the European Union standards for the care and use of experimental animals.

### Mouse and rat TCs isolation and cultivation

The procedures used to isolate TCs from myocardial tissue and subsequently culture them were previously described for mice and rats 2,39,40. Briefly, the hearts of adult C57B6 mice were treated with 1000 U/kg heparin (Sigma-Aldrich, St. Louis, Mo, USA), dissected under a stereomicroscope and mechanically minced into small pieces of approximately 1 mm^3^, followed by enzymatic dissociation. The tissue fragments were incubated for 15 min. on a rocking table in 250 U/ml collagenase II (Sigma-Aldrich) at 37°C; the supernatant, which contained the cells, was collected, and the collagenase activity was inhibited with ice-cold Hank’s Balanced Salt Solution (HBSS). This procedure was repeated twice. The suspension, which contained the dissociated cells was washed and centrifuged, and the cells were re-suspended in DMEM/F12 medium (Sigma-Aldrich) that was supplemented with 10% foetal bovine serum (FBS, Gibco, Paisley, UK) and 100 U/ml penicillin - 100 μg/ml streptomycin (Sigma-Aldrich). The resulting cell suspension was plated in 25 cm^2^ Petri dishes in DMEM/F12 medium that was supplemented as above and then cultivated in an incubator at 37°C in a humidified atmosphere that contained 5% CO_2_. The medium was changed every 24 hrs for the next 7 days or until the cells covered more than 80% of the culture plate. Similar conditions were applied to isolate and culture rat myocardial TCs. The cells were dissociated with trypsin-ethylenediaminetetraacetic acid, washed and centrifuged, re-seeded in DMEM-F12 that contained 10% FBS in 25 cm^2^ flasks and cultivated as described above for passages 2 and 3 at a cell density of 5 × 10^3^ cells/cm^2^. Similar cultures were maintained in FBS-supplemented or serum-free medium for 72 hrs, and the supernatants were collected for analysis. Serum-free medium was used to avoid interference from the FBS in the surface-enhanced laser desorption/ionization time-of-flight mass spectrometry (SELDI-TOF MS) and ‘on-chip’ electrophoresis analyses. The fibroblast cell line BALBc/3T3 (ECACC No. 85022108) was used as a control for cell secretion.

### CSCs culture

The rat CSCs were a generous gift from Prof. Piero Anversa (Brigham and Women’s Hospital, Boston, MA, USA).

### Co-culture of TCs and CSCs

Cell suspensions that were obtained from rat cell cultures were plated at 5 × 10^3^ cells/cm^2^ in 24 mm Transwell cell culture inserts with a 0.4 μm pore clear polyester membrane placed in 6-well plates that contained CSCs 5 × 10^3^ cells/cm^2^ (kindly provided by Prof. Piero Anversa from Brigham and Women’s Hospital) in F12K culture medium. This system permits the free passage of molecules but restricts physical contact between the two cell populations 61–63.

Control wells contained only stem cells in the lower compartment or TCs in the upper compartment. The medium was collected after 48 hrs and stored at −20°C.

### On-chip electrophoretic analysis of proteins

The serum-free supernatants from mouse TCs and 3T3 cell cultures were separated with on-chip electrophoresis using the High-Sensitivity – Protein 250 kit on an Agilent 2100 Bioanalyzer (Agilent Technology, Santa Clara, CA, USA) according to the manufacturer’s instructions 64. The kit contained the following required reagents: molecular weight ladder, fluorochrome, DMSO, 10× Protein-Labeling Buffer (pH 8.0), ethanolamine, gel and destain solution. Briefly, 5 μl of protein samples (including the ladder) were added to 0.5 μl of Protein-Labeling Buffer and labelled with fluorochrome (0.5 μl) for 30 min. on ice. Ethanolamine was added to each reaction mixture, and the excess of fluorochrome was allowed to react for 10 min. on ice. Subsequently, 1 μl of each labelled protein was diluted and further analysed; the samples were stored at −20°C for up to 30 days. For analysis, 1 μl of protein was diluted 200-fold in ultrapure water, and 12 μl of this dilution was then placed into the wells of the bioanalyzer chip. The proteins were separated and the data were analysed using specific software: Agilent 2100 Expert from Agilent Technology.

### SELDI-TOF-MS protein profiling

The cell culture supernatants from mouse TCs and BALBc/3T3 cells were analysed in triplicate on a weak cation-exchange (CM10) ProteinChip array (Bio-Rad Laboratories, Hercules, CA, USA). The chips were prepared and the samples were loaded according to the manufacturer’s instructions for low-stringency buffer conditions. The concentrations of all samples were equalized by adding binding buffer (0.1 M Sodium acetate, pH 4.0), and 1 μl of saturated energy absorbing molecule solution (sinapinic acid in 50% acetonitrile, 0.5% trifluoroacetic acid and 49.5% high-performance liquid chromatography grade water) was added twice and allowed to air dry.

The samples were analysed using time-of-flight mass spectrometry on a PBS II Protein Chip Reader (Bio-rad Laboratories, Hercules, CA, USA). The spectra were collected using the low-setting protocol: focus mass 5000 D, matrix attenuation 1000 D, sampling rate 800 MHz, energy 5000 nJ. The spectra were analysed using the ProteinChip Data Manager Software version 3.0.7 (Bio-rad Laboratories, Hercules, CA, USA). To improve the detection, the protein concentration was enhanced by freeze-drying the samples and reconstituting them in MilliQ ultrapure water (25% of the original volume).

### Luminex-xMAP array multiplex analysis

The cytokine/growth factor levels in the supernatants from serum-free and serum-supplemented mouse TCs and 3T3 fibroblast cell cultures were analysed in triplicate using xMAP technology. The cytokines secreted by mouse TCs were analysed on a Luminex200 (Luminexcorp, Austin, TX, USA) using MILLIPLEX MAP Mouse Cytokine/Chemokine Panel-9 plex – IL-1β, IL-2, IL-4, IL-6, IL-12, tumour necrosis factor (TNF)-α, VEGF, MIP-2 (CXC motif chemokine 2) and granulocyte-macrophage colony stimulating factor (GM-CSF). An enhanced panel was used to analyse the secreted molecules in the rat TC and CSC experiment: MILLIPLEX MAP Rat Cytokine/Chemokine Magnetic Bead Panel, which included G-CSF, Eotaxin, GM-CSF, IL-1β, Leptin, MIP-1α, IL-4, IL-1α, IL-6, epidermal growth factor (EGF), IL-13, IL-10, IL-12p70, interferon (IFN)-γ, IL-6, IL-17, MCP-1, IP-10, GRO/KC, VEGF, Fractalkine, lipopolysaccharide induced CXC chemokine (LIX), MIP-2, TNF-α, IL-2, IL-18, and RANTES. These kits, all from Millipore, Billerica, MA, USA. were used according to the manufacturer’s specifications 65,66.

Briefly, the beads, which were provided within each kit, were incubated with buffer, cytokine standards (included in the kit) or samples in a 96-well plate at 4°C overnight in triplicate. All further incubations with detection antibodies and streptavidin - R-Phycoerythrin Conjugate (SAPE) were performed at room temperature in the dark with shaking at 500 r.p.m. Millipore multiscreen plates were used together with the Millipore filtration system. Multiplex data acquisition and analysis were performed using STarStation software version 2.3 (Applied Cytometry Systems, Sheffield, UK); the calibration curves were generated with a 5-parameter logistic fit. The data are presented as the mean ± SD of three experiments, and two-tailed *P*-values of less than 0.05 were considered to indicate significant differences using Student’s *t*-test.

## Results

### Protein secretory profile of mouse TCs

**Figure 1:**
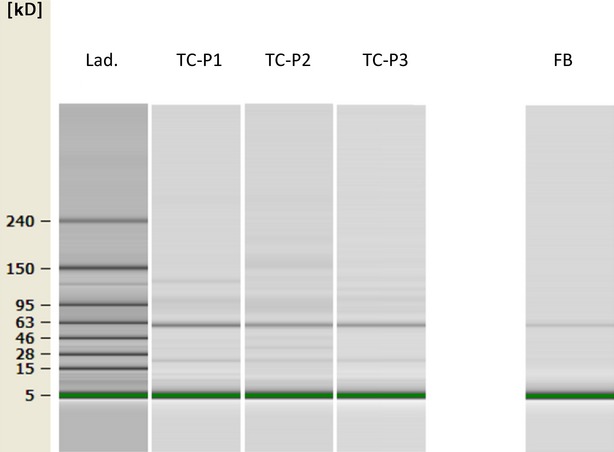
Protein separation of cell-free supernatants from cultures (three subsequent passages) of mouse TCs by on-chip electrophoresis. The bands at 10.3, 13.2, 22.5, 119.2 and 129.5 kD were present in all mouse TCs passages, but not in FB culture. TC-P1 – first passage 1, TC-P2 – second passage, TC-P3 – third passage, FB – fibroblasts, Lad – ladder that illustrates the molecular weight markers.

On-chip electrophoretic analysis consistently revealed the same protein bands throughout all passages of mouse TCs (in cultures that were maintained in serum-free medium), indicating stability between passages. The overall electrophoretic profile is shown in Figure1. Five bands were identified in the supernatants of TCs using the 2100 Expert Software v.B02 from Agilent at molecular weights of 10.3, 13.2, 22.5, 119.2 and 129.5 kD. These bands were not identified in the 3T3 fibroblast cultures that were used as controls. The concentration of the 22.5 kD protein positively correlated with the passage, and this protein band likely is IL-6 and/or VEGF.

**Figure 2:**
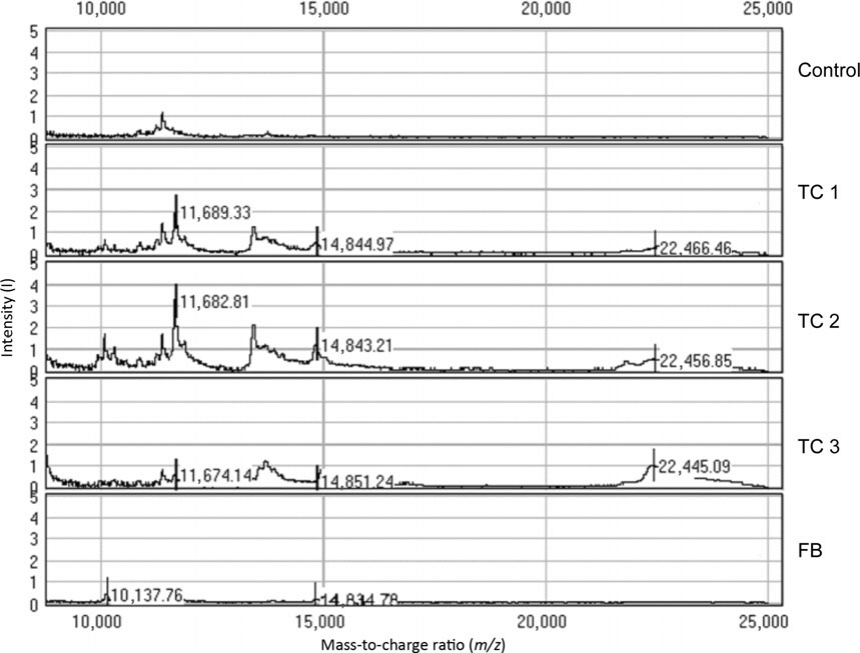
Protein profiling analysis of TC and FB supernatants by surface-enhanced laser desorption and ionization mass spectrometry. Characteristic proteins that are secreted by TCs were identified at m/z values of ∼11.7 kD, from 13.8 to 14.8, and at 22.5 kD. The peaks suggest the presence of MIP-1α, MIP-2 dimer, MCP-1 and VEGF. m/z range: 0–25,000 D, CM10 chips, SPA matrix. Control – cell culture medium, TC-P1 – first passage, TC-P2 – second passage, TC-P3 – third passage, FB – fibroblasts.

SELDI-TOF MS protein profiling was used to analyse and compare several differentially expressed peptide and protein peaks that were identified in the mouse TC culture supernatants, the culture medium and the 3T3 fibroblast culture supernatants. Additionally, several differentially expressed peptide and protein peaks in TCs supernatants (maintained in serum-free medium) were demonstrated by MS. Mass peaks between 1.5 and 25 kD were analysed and the most significant peaks were estimated at m/z values of 11.7 estimated to be most significant. Significant peaks were identified between 13.8 and 14.8 and at 22.5 kD (Fig.2). Using the UniProtKB database 67, the 22.5 kD protein was assigned to the classical family of cytokines/growth factors, and this protein most likely is VEGF or IL-6. Other protein peaks caught our attention, such as those that possibly matched MIP-1α, MIP-2 dimer and MCP-1.

**Figure 3:**
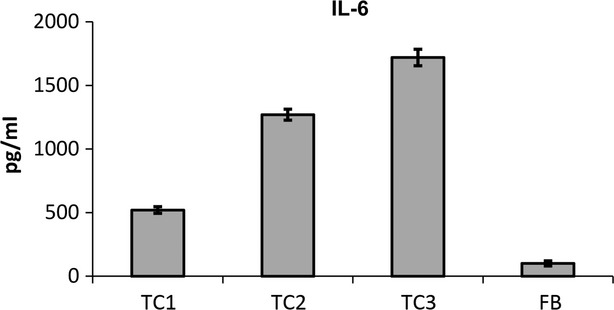
The levels of IL 6 cytokine in the serum-free culture supernatants of mouse myocardial TCs compared to 3T3 fibroblasts as determined by xMAP technology. The data are expressed as the mean ± SE from three independent experiments and were analysed with Student’s *t*-test. TC1 – first passage mouse TCs, TC2 – second passage mouse TCs, TC3 – third passage mouse TCs, FB: fibroblasts.

**Figure 4:**
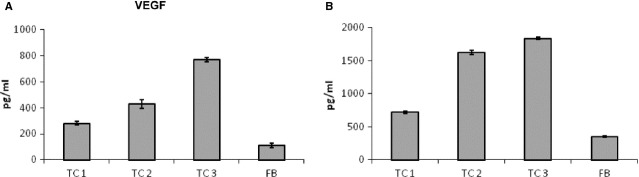
VEGF expression levels in the cell culture supernatants of mouse myocardial TCs compared with 3T3 fibroblasts. (A) VEGF levels in serum-free supernatants. (B) VEGF levels in serum-supplemented media. TC1 – first passage TCs, TC2 – second passage TCs, TC3 – third passage TCs, FB – fibroblasts.

To precisely identify the detected proteins, we further used xMAP analysis to examine the protein composition of mouse TCs supernatants. We identified IL-6, VEGF and MIP-2, but no other inflammatory cytokines or mediators, such as TNF-α, IL-1β, IL-2 or IL-4, were detected (Figs3 and 4). Table1 shows the synthesis of the secretome of mouse TCs with additional data that were extracted from the UniProtKB database 67.

**Table 1:** Proteins that were identified in mouse TC culture supernatants by Luminex-xMAP and their main characteristics obtained from the UniProtKB database

Molecule	Name	Alternative names	Molecular weight	UniProtKB code	Roles	Concentration, pg/ml*
MIP-2	CXC motif chemokine 2	Macrophage inflammatory protein 2	7849.31	P10889	Chemotaxis, cell migration	155 ± 27
IL-6	Interleukin-6	IL-6	21,734.75	P08505	Positive regulation of ERK1 and ERK2 cascade Cytokine, Growth factor	1270 ± 43
VEGF	Vascular endothelial growth factor	VEGFA, VEGF	22,121.52	Q00731	Growth factor, Mitogen	1630 ± 32

* Concentrations given for TC–passage 2.

The mentioned proteins were specifically detected in mouse TCs supernatants, but not in the serum-free culture medium (used to replace the medium containing FBS 24 hrs before supernatant harvesting). Mouse TCs secreted significantly more IL-6 than fibroblast cultures in both serum-free and -supplemented media. In serum-supplemented medium, mouse TCs secreted more than threefold the level of IL-6 than 3T3 fibroblasts (*P* < 0.05), and this level did not significantly differ by passage. Cells grown in serum-free medium secreted significantly more IL-6 (8- to 19-fold increase) than the corresponding 3T3 culture. Mouse TCs secreted significantly more proteins in serum-free conditions than 3T3 fibroblasts (fourfold increase in mouse TCs at first passage (P1) compared with fibroblasts at the same passage, *P* < 0.05), and this secretion level increased the most (almost 10-fold) in the third passage (P3) (*P* < 0.01; Fig.3).

VEGF secretion followed a similar pattern: in serum-free medium, TC secretion increased 1.9-fold in the first passage (P1), threefold in the second passage (P2) and 5.5-fold in the third passage (P3) compared with the fibroblast culture. In serum-supplemented medium, TCs secreted 1.5-fold more VEGF in P1 than fibroblasts. Moreover, TCs secreted significantly more VEGF in P2 than in P1 (3.3-fold, *P* < 0.05), and this level increased further (3.8-fold, *P* < 0.05) in the TCs of P3 (Fig.4).

The positive correlation between the secreted levels of IL-6 and VEGF and the passage number suggests a possible autocrine stimulation of TCs when cultivated in serum-free medium; analog stimulatory molecules that are present in FBS may ‘mask’ the presence of these molecules under serum-supplemented conditions.

### Rat cardiac TCs and CSCs secretomes and their reciprocal influence

The secretomes of cultivated myocardial TCs, CSCs and a co-culture of TCs and CSCs from rats were compared. The secretory profile of several cytokines and chemokines in the cell culture supernatants was evaluated using SELDI technology and xMAP methods, and an enhanced panel of cytokines-chemokines, including IL-2, IL-5, IL-6, IL-10, VEGF, MCP-1, MIP-1α, MCP-2, GRO/KC and IL-13, was identified.

**Figure 5:**
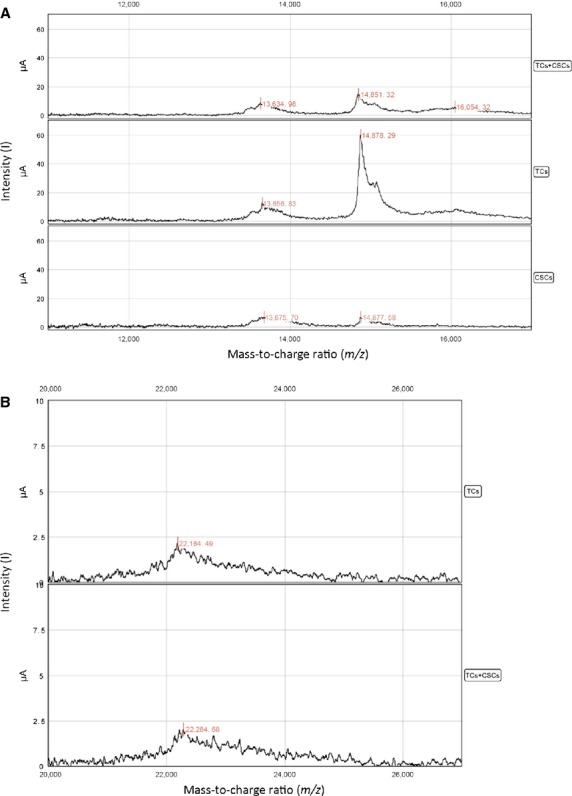
(A) Proteins that were secreted in the cell culture supernatants of rat myocardial TCs as assessed by SELDI-TOF-MS analysis. A significant enhancement of the 14.8 kD peak in TC mono-culture compared with CSC mono-culture and TC-CSC co-culture is evident. m/z range: 10,000–20,000 D, CM10 chips, SPA matrix. (B) Outline of the 22.2 kD peak (IL-6) in the SELDI-TOF-MS spectra of proteins that were secreted by rat TCs. CM10 chips, SPA matrix. The expression of the protein associated with this peak does not significantly differ between the TC and TC-CSC co-cultures.

SELDI-TOF profiling identified several protein peaks in the culture medium supernatants from rat TCs, rat CSCs or TCs-CSCs co-cultures grown under serum-free conditions. The most significant differentially expressed peaks were at 7.5, 8.5, 13.6, 14.9, 22.3, 27.5 and 33.4 kD. This result is consistent with the findings for mouse TCs. Two regions of SELDI-TOF MS spectra are shown: Figure5A illustrates the portion of the mass spectrum of proteins between 10 and 17.5 kD, which outlines the presence of a significantly expressed protein of m/z = 14.7 kD (likely MCP-1). Figure5B shows a peak at 22.3 kD (likely IL-6). IL-6 was identified in the supernatant of rat TCs, which correlates with the mouse TC secretome; in the rat TCs-CSCs co-culture system, IL-6 was expressed at levels similar to those in the rat TCs culture.

Using the Luminex-xMAP analysis, we identified and quantified several molecules and compared their expression in the TCs, CSCs and co-culture supernatants. Luminex xMAP is very specific (the cross-reactivity for molecules from other species or for related molecules is less than 0.5%). This method permitted the highly accurate detection and quantitation of proteins in supernatants of cells that were cultivated in normal (FBS-supplemented) medium.

**Figure 6:**
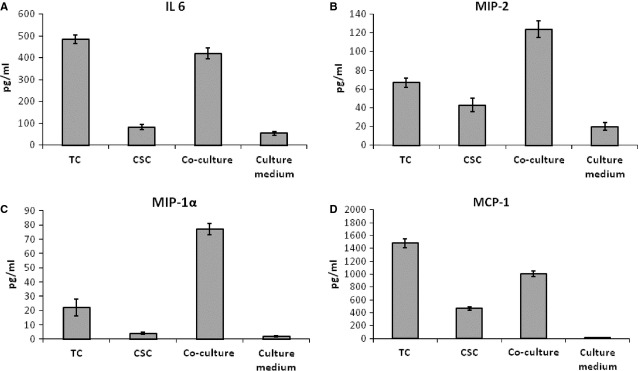
Luminex-xMAP detection of secreted cyto- and chemokines in the cell culture supernatants of rat myocardial TC mono-, CSC mono- and TC-CSC co-cultures: (A) IL-6, (B) MIP-2, (C) MIP-1α, (D) MCP-1. The results were normalized to cell counts. *P* < 0.05, *n* = 3.

The rat secretome contained significantly more protein than the CSC secretome. IL-6 was significantly expressed in the rat secretome at levels similar to those in the mouse TC secretome, *i.e*., TCs expressed a fivefold higher level than CSCs (Fig.6A). This concentration remained high and similar to the TC secretome level in the TC-CSC co-culture supernatant.

The levels of VEGF, MCP-1 and GRO-KC secreted by TCs and CSCs were relatively similar. The expression levels of several molecules were modified in response to TCs-CSCs co-culture.

Co-culture increased the level of MIP-2 by twofold compared with TC mono-culture and threefold compared with CSC mono-culture. The TC mono-culture supernatant contained twofold more MIP-2 than the CSC mono-culture supernatant (Fig.6B). This effect is due to the increase in IL-6, because IL-6 and IL-6R induce the expression of MIP-2.

The rat TC culture supernatant contained high levels of VEGF, which is consistent with the levels measured in mouse TC cultures. The VEGF levels were slightly lower in rat CSC mono-culture and TC-CSC co-culture. This finding indicated that the expression of VEGF is not cross-modulated in TC-CSC co-culture.

In TC-CSC co-culture, the expression of MIP-1α (CXCL 3) was threefold higher than in TC and 20-fold higher than in CSC mono-culture (Fig.6C).

Although IL-2 was well-expressed in the individual rat TC and rat CSC cultures, it appeared to be negatively regulated in the TC-CSC co-culture (approximately twofold reduction compared with mono-culture).

Consistent with the secretory profile of MIP-2, we found MCP-1 to be highly expressed in TC culture (threefold higher than in CSC culture), and this protein was also expressed at high levels in the rat TC-CSC co-culture (twofold higher than in CSC culture; Fig.6D).

The secreted levels of GRO/KC (CXCL 1) were high in the TC monoculture, moderate in the TC-CSC co-culture and low in the CSC mono-culture supernatants.

Interleukin-10, an important anti-inflammatory molecule, was significantly expressed in all cultures (TC, CSC and TC-CSC co-culture), while IL-5 was moderately expressed in mono-culture (TC and CSC) and unaffected by the co-culture conditions.

The expression of IL-13 was up-regulated in the TCs-CSCs co-culture compared with the TC mono-culture.

The rat cardiac TCs data were consistent with the mouse TC data. Luminex-xMAP identified a larger array of proteins than the entries in the UniProtKB database 67. A summary of these molecules and the main protein information are given in Tables1 and 2.

**Table 2:** Proteins that were identified in rat TC and CSC culture supernatants using Luminex-xMAP

Protein	Name	Alternative names	Molecular weight	UniProt KB code	Roles	Concentration, pg/ml	
						TCs	CSCs
MIP-2 rat CXCL2 rat	CXC motif chemokine 2	Macrophage inflammatory protein 2	31,307.36 (tetrameric)	P30348	Chemotaxis	67 ± 4.9	43 ± 6.2
IL-6 rat	Interleukin-6	IL-6	21,732.90	P08505	Positive regulation of ERK1 and ERK2 cascade Cytokine, Growth factor	485 ± 19	82 ± 11
VEGF rat	Vascular endothelial growth factor	VEGFA, VEGF	22,077.47	P16612	Developmental protein, Growth factor, Mitogen Angiogenesis, Differentiation	475 ± 31	304 ± 24
MIP-1α	C-C motif chemokine 3	Macrophage inflammatory protein 1-alpha MIP-1α	7853.90	P50229	Chemotaxis, Inflammatory response	22 ± 5.6	4 ± 0.8
IL-2 rat	Interleukin-2	IL-2	15,493.97	P17108	Positive regulation of tyrosine phosphorylation of Stat5 protein	14.2 ± 0.9	13.5 ± 0.7
IL-5 rat	Interleukin-5	IL-5	13,074.22	Q08125	Positive regulation of cell proliferation	69 ± 1.9	18 ± 2.6
IL-13 rat	Interleukin-13	IL-13	12,075.14	P42203	Positive regulation of connective tissue growth factor production	7.2 ± 0.6	12.3 ± 0.8
					Negative regulation of neuron death		
MCP-1 rat	Monocyte chemoattractant protein 1	C-C motif chemokine 2	14,066.11 (dimer)	P14844	Cellular response to fibroblast growth factor; organ regeneration	1490 ± 55	467 ± 36
	Monocyte chemotactic protein 1				Cellular response to macrophage colony-stimulating factor;		
GRO-KC rat CXCL1	Growth-regulated alpha protein	C-X-C motif chemokine 1	7849.37 × 2	P14095	Cytokine, Growth factor	940 ± 41	539 ± 54
IL-10 rat	Interleukin-10	IL-10	18,640.56	P29456	Negative regulation of inflammatory response, inhibition of apoptosis	18 ± 1.7	13 ± 2.1

## Discussion

This study primarily aimed to assess the protein/peptide secretory profile of TCs from rodent hearts. The results reported herein show that the levels of several cyto- and chemokines (IL-6, MIP-1α, MIP-2 and MCP-1), as well as VEGF, were significant in mouse and rat TC supernatants. These findings suggest the potential regulatory effect of TCs on other cell types (myocardial stem and myocardial progenitor cells) and their role in the control of cell growth/myocyte differentiation and angiogenesis. However, further advanced proteomics studies or antibody arrays are needed to confirm these findings.

Recent studies have shown that TCs actively participate in intercellular communication over long distances *via* Tps and over short distances *via* EVs 3,56. Furthermore, proteomic studies identified several proteins that were up-regulated in both TCs and EVs, which suggests that these proteins play a role in intercellular signalling and stem cell niche modulation 43,44. The latest reviews emphasize the importance of exosomes in normal and pathologic hearts 68, as well as in regenerative medicine 69. This importance depends on the origin of the exosome. Few studies provide evidence for paracrine cross-talk between cardiac cell populations *via* EVs, which transport crucial cardioprotective agents 57,70. Moreover, exosomes that are derived from the ischaemic preconditioned myocardium, mesenchymal stem cells (MSCs) and cardiac progenitor cells provide cardioprotection, while exosomes that are derived from hematopoietic stem cells promote angiogenesis in the myocardium 68. In addition, TCs release and internalize EVs that are loaded with microRNAs and epigenetically control cardiac stem and progenitor cells 9.

The IL-6 cytokine family includes LIF, CT-1 and IL-11, which activate downstream signalling pathways in cardiac myocytes and contribute to cytoprotection and vessel formation in the heart 71. Interleukin-6 stimulates the production of VEGF by MSCs; thus it contributes to cardio-protection during myocardial ischaemia 71. We found enhanced levels of IL-6 in mouse TC culture supernatants that might represent a regulatory factor in cardiac angiogenesis and regeneration. Interleukin-6 was also detected in rat TC culture and in TC-CSC co-culture experiments, together with several other chemokines that were induced by IL-6 signalling 67,71, such as MIP-1α, MIP-2 and MCP-1, and are involved in cell proliferation. Moreover, the estimated secretory profiles of IL-6 and VEGF under serum-depleted conditions indicate a possible autocrine stimulation that most likely depends on IL-6. Although IL-6 is a pro-inflammatory molecule, it is not regulated by TNF-α, suggesting that the expression of this cytokine remains controlled as a gradient. This gradient induces the expression of regenerative molecules, because an array of factors intervenes at distinct stages in the complex cardiac remodelling process 72. Interleukin-6-type cytokines are involved in cardiac repair *via* the engagement of the JAK-STAT3 axis. *In vitro*, cytokines activated JAK-STAT3 signalling and increased the expression of the STAT3 target genes hepatocyte growth factor and VEGF 73. *In vivo* studies showed that JAK-STAT3 signalling was activated and growth factor/cytokine production was increased in MSC-injected animals. The paracrine actions of these host tissue-derived factors activated the endogenous cardiac repair mechanisms in the diseased heart mediated by Akt, ERK and JAK-STAT3 73.

The levels of several secreted molecules are higher in rat TCs than in CSCs, *e.g*. IL-6, MIP-1α, MIP-2, MCP-1 and IL-5, which suggests that TCs play a role in paracrine regulation. In addition, the co-culture experiments suggest that TCs and CSCs act synergistically to control the secretion of proteins, as illustrated by the levels of MIP-1α, MIP 2 and IL-13 (up-regulated) as well as the level of IL-2 (significantly down-regulated). As expected, the levels of these chemokines are up-regulated, because they are regulated by IL-6 74. The response of the co-culture (increased expression of GRO/KC) is consistent with other *in vivo* findings of the role of TCs in myocardial regeneration.

We also detected high levels of VEGF in the TC mono-culture and TC-CSC co-culture experiments. Tang *et al*. showed that VEGF secreted by MSCs improves myocardial survival and the engraftment of implanted MSCs in infarcted hearts, which promoted the recruitment of stem cells *via* the paracrine release of myocardial stromal cell-derived factor-1α 75. The expression of Flk-1, a VEGF receptor, was induced by IL-6-family cytokines in cardiac Sca-1+ cells 71. Therefore, the presence of VEGF and IL-6 in TC supernatants suggests a potential regulatory role of TCs in the control of cell growth/myocyte differentiation and angiogenesis. This hypothesis is consistent with previous findings regarding the implication of these cells in promoting post-myocardial infarction angiogenesis 76.

We correlated the secretion of VEGF by TCs reported herein with a report by Song *et al*., who demonstrated the role of VEGF in the differentiation of stem cells into cardiomyocytes 77. VEGF is a paracrine mediator of MSC-mediated cardiac protection 78. The delivery of MSCs into the ischemic myocardium increases local VEGF levels and thereby improves myocardial function 79–81. MSCs that are engineered to overexpress myocardial protective factors, such as VEGF and protein kinase B (Akt), play a role in improving survival by enhancing cardiac protection, likely by vascularizing functional tissue constructs 82. Mesenchymal stem cells constitutively secrete high levels of IL-18BP (IL-18 binding protein), which are associated with high levels of VEGF 83. The presence of VEGF in TC supernatants might be of extreme importance, because the positive effect of growth factors administered *via* delivery systems in regenerative medicine is well-known 68,84. Therefore, we can theorize that TCs may also exert increased cardioprotective effects *via* bi-directional paracrine cross-talk with CSCs, an additional argument for the potential application of TCs in myocardial regeneration. In addition, the co-culture experiments demonstrated the bi-directional modulation of TCs and CSCs, as demonstrated by the modification of the secretory profile, which indicated that MCP-1 and MIP-1α production were enhanced in co-culture compared with TC or CSC mono-culture. These findings suggest that these chemokines may mediate the role of TCs in directing the formation of cardiomyocytes. MIP-1α and MCP-1 were shown to play similar roles in the formation of smooth muscle in the airway 85,86. Additionally, MCP-1 appears to be involved in mouse skeletal muscle regeneration 87 by recruiting macrophages. The enhancement of MCP-1 secretion, as identified herein in TC-CSC co-cultures, can serve as activator of another cell population, primarily macrophages, which are generally involved in such processes 88. Mesenchymal stem cells that were transplanted to the injured myocardium shifted the balance of macrophages to the M2-like phenotype 89. Similarly, in a spinal cord injury model, MSC transplantation shifted the macrophage phenotype to an M2-like phenotype, which improved functional recovery 90.

We suggest that TCs, which secrete more growth factors and intercellular communication cytokines than other cell types, can deliver signals to stem cells that drive them to differentiate into myocardial cells and participate in the complex process of myocardial regeneration. Moreover, the heart regeneration process, similar to those of other organs, depends on the integrity of the extracellular matrix, specifically that of fibronectin 91. Fibronectin is a prerequisite for pluripotent cell self-renewal decisions in the stem cell niche 92. High levels of fibronectin determine the differentiation of cells and the loss of the self-renewal markers Nanog and Oct4 93. Furthermore, TCs were previously shown to adhere and spread better than fibroblasts *in vitro* 40. Taken together, these findings suggest that TCs synergistically act with stem cells to affect heart regeneration. Moreover, the interplay between inflammatory cytokines/chemokines sustains a delicate equilibrium between pro-inflammatory (IL-6 and KC/GRO) and anti-inflammatory (IL-5 and IL-13) cytokines, which drives cells towards regeneration. Because these molecules were identified in the secretome of cardiac TCs, we can once more support the belief that TCs play an important role in influencing the cardiac stem cell fate *via* paracrine intercellular signalling.

## Conclusion

We report here, for the first time, the secretome of cultivated TCs that were isolated from rodent hearts. These data further support the shuttle mechanism of vesicle-mediated signalling between stem cells and TCs. The secreted forms of some chemokines, cytokines and growth factors identified in TC primary cultures suggest that these secretory molecules may be useful in cardiac repair. The panel of cytokines/chemokines identified in this study suggests that IL-6 drives cells towards a proliferative phenotype. In-depth studies of the future therapeutic applications of TCs should focus on mechanisms that control the intercellular communication. Thus, TCs could be key players in the renewal and repair of some organs, including the heart, and TC-CSC co-culture may be a superior option for therapy *versus* CSC mono-culture.
